# Project Soothe: A pilot study evaluating the mood effects of soothing images collected using a citizen science approach

**DOI:** 10.12688/wellcomeopenres.18950.2

**Published:** 2024-03-01

**Authors:** Keren MacLennan, Matthias Schwannauer, Angela L. McLaughlin, Stephanie Allan, Simon E. Blackwell, Fiona Ashworth, Stella W. Y. Chan

**Affiliations:** 1 Department of Psychology, Durham University, Durham, UK; 2 School of Psychology and Clinical Language Sciences, University of Reading, Reading, UK; 3 School of Health in Social Science, University of Edinburgh, Edinburgh, UK; 4 School of Health and Wellbeing, University of Glasgow, Glasgow, UK; 5 Clinical Psychology & Psychotherapy, Universitätsklinik der Ruhr-Universität Bochum, Bochum, Germany; 6 St. George’s Healthcare NHS Trust, London, UK

**Keywords:** self-compassion, psychotherapy, mental imagery, depression, wellbeing, citizen science

## Abstract

**Background:**

Mentally-generated soothing imagery is a therapeutic technique to support mental wellbeing, but some individuals may require support using externally presented stimuli. Project Soothe was launched to collect soothing images using a citizen science approach. This online pilot study evaluated the first 575 soothing images collected, examining: 1) if the images were perceived to be soothing; 2) if viewing the images had a positive impact on mood; and 3) if mood effects were influenced by individual differences in age, gender and depressive symptoms.

**Methods:**

We recruited 1152 participants (13 – 79 years, M = 35.62, SD = 14.60; 77% female). Participants were randomly allocated to one of 23 sets, each containing 25 images (n = 50 per set) and asked to rate their emotional response (soothed, excited, and anxious) to each image. Participants also reported their mood states pre- and post-viewing the images (using the International Positive and Negative Affect Schedule – Short Form).

**Results:**

Project Soothe images were rated to be significantly more soothing than anxiety- or excitement-inducing. Further, viewing 25 images was significantly associated with an increase in positive affect and decrease in negative affect. These effects were associated with age and depressive symptoms, with older individuals and those with lower depressive symptoms being associated with more positive changes in mood.

**Conclusions:**

This pilot study provides preliminary evidence that these soothing images can promote positive mood changes. Further work can now replicate these findings in larger-scale studies with comparison groups and extended outcome variables. The images and associated data have been made available in a data repository (OSF) as a free resource for researchers and practitioners. It is hoped that these images can be developed into useful therapeutic resources.

## Introduction

Through our research and public engagement programme ‘Project Soothe’ ( projectsoothe.com), we have piloted a Citizen Science methodological approach to collate a bank of soothing photographs submitted by the public. At the time of this study, we have collected over 500 soothing images from the members of the public from more than 30 countries. We specifically chose to use a citizen science approach as there has been growing evidence to suggest that, in addition to achieving our research goals, it offers the added value of enhancing public’s awareness and knowledge of science (in this case, health and mental health) ( Bonney *et al.*, 2016). We further chose to collect photographs that activate ‘soothing’ feelings as ‘soothe’ has been proposed in Gilbert’s three-circle model of emotion ( Gilbert, 2009) to be a key affective system that balances the other two affective systems of ‘threat’ and ‘drive’. In this theoretical context, the threat system was proposed to denote feelings associated with a threat response such as fear, anxiety and anger, while the drive system embodies the motivation to achieve and is associated with feelings such as excitement and anticipation. By contrast, feelings of soothe, characterised by interconnected feelings of ease, relaxation and calmness, cultivated by safeness, contentment, and inner peace ( Mok *et al.*, 2020), have been associated with decreased self-criticism and increased compassion and self-compassion ( Gilbert, 2009; Gilbert *et al.*, 2006; Gilbert & Irons, 2004; Judge *et al.*, 2012). It has been proposed that mental health problems can arise if these systems become unbalanced. Therapeutic techniques, such as those used in compassion-focused therapy (CFT), have therefore been developed to support individuals to activate soothing feelings to help rebalance these innate systems and promote positive mental wellbeing ( Gilbert, 2009; Gilbert, 2014). Our groundwork suggested that the everyday experiences of soothing feelings resonate with that proposed in theory ( Mok *et al.*, 2020), and that emotional responses to externally presented Project Soothe images are similar to mentally generated positive imagery ( Wilson *et al.*, 2018). We have further demonstrated that both soothing images and soothing sounds reduce negative mood states and increase positive mood states ( Witten *et al.*, 2023). While these preliminary findings suggest that Project Soothe images have the potential to be developed into clinically useful resources to boost mental wellbeing, we need to first validate the images by testing if these images elicit the type of emotional responses that they were intended to.

In particular, the Project Soothe images could be used therapeutically to support existing mental imagery approaches. Mental imagery, characterised by representations and associated sensory stimuli recalled from memory with the absence of direct external stimuli ( Pearson *et al.*, 2015), is thought to be associated with emotion states ( Holmes & Mathews, 2010). It plays an important role in many different psychopathologies, for instance, post-traumatic stress disorder and social phobia ( Hirsch & Holmes, 2007), where concern-related imagery is believed to be involved in the development and maintenance of these conditions ( *e.g.*, Ehlers & Clark, 2000). This interconnection between imagery and emotion implies that stimulating mental imagery can have a positive impact on mood. Consistent with this, imagery evoked from positive picture-word cues has been shown to lead to increases in positive affect ( Pictet *et al.*, 2011), and interventions involving the generation of positive imagery have appeared to be potentially beneficial for depression ( Blackwell, 2021). In particular, individuals who experience greater vividness in mental imagery were reported to benefit from a greater reduction of depressive symptoms ( Blackwell *et al.*, 2015). This implies that individuals who struggle with generating mental imagery may require additional support to benefit from this strategy in therapies. Therefore, one strategy to assist those who struggle to produce mental imagery could be to use positive external stimuli, as it is thought that this could effectively support individuals to upregulate their mood ( Gilbert, 2007; Hackmann *et al.*, 2011; Singer, 2006). These hypothesized effects are consistent with Lang’s (1979) bioinformational theory, which proposed that similar cognitive networks underline processing of positive external stimuli and positive mental imagery, stimulating similar emotional responses. Indeed, research has suggested that externally presented images can be a reliable ‘sensory scaffold’ to stimulate episodic simulation and also user engagement in cognitive training to reduce anxious thinking ( Ji *et al.*, 2020). However, limited research has so far examined the effects associated with using positive externally presented stimuli, such as photographs.

Within the theoretical context discussed above, the primary objective of this research was to test if Project Soothe images elicit soothing feelings rather than the contrasting feelings associated with the affective systems of threat and drive. As a proof-of-concept study, our secondary aims were to examine if viewing the images would induce immediate positive mood changes and explore if the findings would vary with individual differences in demographic and clinical characteristics (including age, gender, and level of depressive symptoms). Based on existing literature and our groundwork above, it was hypothesised that participants would feel significantly more soothed than anxious or excited in response to viewing the images, and that viewing Project Soothe images would result in an increase in positive affect and decrease in negative affect. The final aim was investigated for exploratory purposes as there have not been sufficient previous research to formulate hypotheses around individual differences.

## Methods

### Ethics approval statement

This study obtained ethical approval from the Department of Clinical and Health Psychology Research Ethics Panel at the University of Edinburgh and the School of Psychology and Clinical Sciences Research Ethics Panel at the University of Reading.

### Participants

The sample included 1152 participants aged 13 – 79 years (Mdn = 32 IQR = 21; M = 35.62, SD = 14.60), with 892 (77.43%) participants self-identifying as female, 233 (20.23%) as male, 20 (1.73%) as other genders, and seven (0.61%) did not disclose their gender. The Patient Health Questionnaire-9 (PHQ-9; Kroenke *et al.*, 2001) indicates that depression scores ranged from 0 to 27 (M = 7.01, SD = 6.04), with 520 (45.14%) having scores below the cut-off for depression (0–4), 307 (26.65%) having scores indicative of mild depression (5–9), 173 (15.02%) having scores indicative of moderate depression (10–14), 87 (7.55%) having scores indicative of moderately severe depression (15–19), and 65 (5.64%) having scores indicative of severe depression (20–27). Participants were recruited via social media (Twitter, Facebook) and listed on websites for Citizen Science projects. Recruitment and data collection was from 12 months, between May 2017 and May 2018. Written informed consent was obtained from all participants. This study obtained ethics approval from the relevant University ethics committee.

### Procedure

This study was conducted online using Online Surveys (formerly Bristol Online Surveys). After reading the information sheet and providing written informed consent, participants completed demographic questions (as reported above). They then completed the International Positive and Negative Affect Schedule – Short Form (I-PANAS-SF; Karim *et al.*, 2011), followed by the image rating task, and then completing the I-PANAS-SF again. Lastly, participants completed the PHQ-9 to index their level of depressive symptoms, which was positioned at the end to avoid the risk that filling in a mood questionnaire may bias the way participants reported their current mood states. Details of the task and questionnaires are described below.

### Materials

***Image ratings task.*** At time of this evaluation study, 575 images had been collected through the Project Soothe website (projectsoothe.com) and were included in the image rating task. Categorisation of these images identified themes relating to water features (151), flowers and trees (112), landscapes (99), animals (75), sky (53), other images (36), people (22), buildings (16), and snow (11), which was determined through consensus coding of the most salient theme of each image. As it was not feasible to ask participants to rate 575 images, prior to the study the research team randomly allocated these images into 23 sets of 25 images. Each set of 25 images was inserted into one of the 23 surveys, which were otherwise identical. Participants were allocated to image sets sequentially. In other words, each image in the whole collection was rated by 50 participants. In this task, participants were asked to view one set of 25 images, with the images presented in the same order for each participant, and rate how ‘soothed’, ‘anxious’, and ‘excited’ they felt in response to looking at each image. These ratings were on a seven-point Likert scale from 1 to 7 (‘not at all’ – ‘very much’), with higher scores relating to stronger affect. The terms ‘soothed’, ‘anxious’, and ‘excited’ were chosen as they are feelings proposed to be associated with the corresponding Soothe, Drive, and Threat affective systems from Gilbert’s theory of compassion ( Gilbert, 2009). The instructions as the commencement of this task were: “You will now be shown a collection of images for you to evaluate. For each image, please take a moment to consider how soothed, anxious and excited the image makes you feel. Then, use the scales under each image to rate your feelings.” Participants were able to view the images an unconstrained amount of time before providing their ratings.

***International Positive and Negative Affect Schedule – Short Form (I-PANAS-SF).*** The I-PANAS-SF ( Thompson, 2007) was used to measure positive and negative affect pre-viewing and post-viewing the 25 images. Participants were asked to rate to what extent they are currently experiencing positive mood states (active, determined, attentive, inspired, alert) and negative mood states (afraid, nervous, upset, hostile, ashamed). These 10 items were rated on a five-point Likert scale from 1 – 5 (‘not at all’ – ‘extremely’). Total scores were calculated for positive affect and negative affect separately, each ranging from 5 to 25, with higher scores reflecting greater positive or negative affect. The I-PANAS-SF has been shown to have acceptable to good internal consistency for positive affect (α = 0.75) and negative affect (α = 0.80) ( Karim *et al.*, 2011). In our sample, we found the I-PANA-SF to have similarly good internal consistency for positive affect (α = 0.75 - 0.84) as well as negative affect (α = 0.85).

***Patient Health Questionnaire – 9 (PHQ-9).*** The PHQ-9 ( Kroenke *et al.*, 2001) was used to assess levels of depression. It is a nine-item self-report questionnaire that measures symptoms of major depressive disorder (MDD) in line with the Diagnostic and Statistical Manual of Mental Disorders ( DSM-5 American Psychiatric Association, 2013). Items were rated on a four-point Likert scale from 0 – 3 (‘not at all’ – ‘nearly every day’) and respondents were asked to rate items based on frequency of experience over the previous two weeks. Total scores range from 0 – 27, with higher scores relating to greater depression severity. The PHQ-9 has been shown to have excellent internal reliability (α = 0.86 - 0.89) and discriminant validity (α = .95) ( Kroenke *et al.*, 2001). In our sample, the PHQ-9 was found to have excellent internal consistency (α = 0.89).

### Analyses

Data processing and statistical analysis were conducted using RStudio ( RStudio Team, 2020) and JASP ( JASP team, 2020). Distribution checks indicated age, depressive symptoms, negative affect, anxious rating, and excited rating were not normally distributed, and non-parametric tests were used where deemed appropriate.

Firstly, to examine the emotional responses to the Project Soothe images (aim 1), we conducted a repeated measures ANOVA to test if participants felt significantly more soothed than anxious or excited from viewing the images, with emotional valence (soothed, anxious, and excited) as the within-subject factor, and including the image set number as a between-subject factor. Mauchly's Test of Sphericity indicated that the assumption of sphericity had been violated, *χ²*(2) =.242.724, p < 0.001, therefore, Greenhouse-Geisser corrected results were reported. The above analysis was based on a sample size of 50 as it relates to the data available for each image ( *i.e.*, the total number of participants assigned to each image set). To inform clinical and research application use of the images we created a spreadsheet with the relevant descriptive data for each image, which are ranked based on their mean rating of soothed from the most soothing to the least soothing.

Secondly, to investigate if viewing 25 of the Project Soothe images resulted in positive mood changes (aim 2), we conducted two separate repeated measures ANOVAs to test if there were changes in positive affect and negative affect across time (pre-viewing images *versus* post-viewing images), again including set number as a between-subject factor. This analysis was based on a sample size of 1152 ( *i.e.*, all participants completed these measures regardless of set number).

Thirdly, we conducted a series of analyses to explore if the above results vary with individual factors, including age, gender, and depression symptoms, (aim 3). These analyses were based on the full sample of 1152. Before collapsing the data across sets, we tested if there were significant differences in demographic characteristics between sets. We examined age using a one-way ANOVA (with age as the dependent variable and set number as the fixed-factor), we examined gender frequencies between sets using a chi-square analysis (only male and female participants were included in the analysis due to small numbers of other genders), and we examined depressive symptoms using a chi-square and a one-way ANOVA (with PHQ-9 score as the dependent variable and set number as the fixed-factor).

To examine age effects, we conducted a Spearman’s bivariate correlation analysis to test if age was related to emotional responses (feelings of soothed, anxious, and excited) from viewing the images. We further conducted two separate repeated measures ANCOVAs to test if changes in positive and negative affect across time (pre-viewing images vs post-viewing images) were replicated when including age as a covariate.

To examine gender effects, we conducted independent-sample Wilcoxon signed-rank tests to compare if mean soothed, anxious, or excited ratings differed between male and female participants. We further conducted two separate 2 (Time: pre-viewing images and post-viewing images) ×2 (Gender: female and male) repeated measures ANOVAs to test if changes in positive and negative affect across time were replicated when including gender as a between-subject factor. Although not all participants identified within a binary gender, we only examined gender effects in females (n = 892) and males (n = 233) due to too small numbers of other genders (n = 20) or those who did not disclose their gender (n = 7).

To examine depressive symptom effects, we conducted a Spearman’s bivariate correlation analysis to test if PHQ-9 scores were related to emotional response (feelings of soothed, anxious, and excited) from viewing the images. We further conducted two separate 2 (Time: pre-viewing images and post-viewing images) × 2 (Depression level: symptomatic and asymptomatic) repeated measures ANOVAs to test if changes in positive and negative affect across time were replicated when including depression level and set-number as between-subject factors. Groups based on level of depressive symptoms were established as symptomatic (n = 632), which included those who scored as above the cut-off for having mild or higher level of symptom severity a on the PHQ-9 (score ≥ 5), and as asymptomatic (n = 520), which included those who scored as having few or no depressive symptoms (score 0–4). We collapsed the categories this way due to the data being from a non-clinical sample and therefore higher levels of symptom severity did not contain sufficient cases to be statistically powered. To further examine the potential relationship with level of depressive symptoms, we supplemented the above by conducting two separate repeated measures ANCOVAs to test if changes in positive and negative affect across time (two levels: pre-viewing images and post-viewing images) were replicated when set number was entered as a between-subject factor and the PHQ-9 scores were entered as a covariate. The utility of examining depressive symptoms from a categorical and continuous perspective is that the PHQ-9 is a measure often used in clinical practice (e.g. IAPT service), where categories are used. Therefore, conducting analyses using categories of symptomatic vs asymptomatic would provide practitioners information that can be more easily interpreted in clinical contexts. At the same time, by categorising scores and collapsing groups like this, we would not be able to provide more precise findings regarding the association with the exact level of depressive symptoms. Therefore, we also performed analyses where PHQ scores were used as a continuous variable.

## Results

### Aim 1: Analyses to test the emotional responses to Project Soothe images

Table 1 reports descriptive statistics for the overall soothed, anxious, and excited ratings for the images. The spread of data (SD and IQR) was larger for soothed ratings than for anxious and excited, indicating larger individual differences in the soothing effect of looking at the images.

**Table 1.** Descriptive statistics for overall soothed, anxious, and excited ratings for the soothing images (n = 1152). SD: standard deviation; IQR: interquartile range.

	*Range*	*Mean*	*SD*	*Median*	*IQR*
**Soothed**	1.34 – 5.52	3.77	0.84	3.88	1.15
**Anxious**	1.12 – 3.28	1.64	0.35	1.54	0.44
**Excited**	1.26 – 3.68	2.15	0.45	2.10	0.61

**Figure 1.**
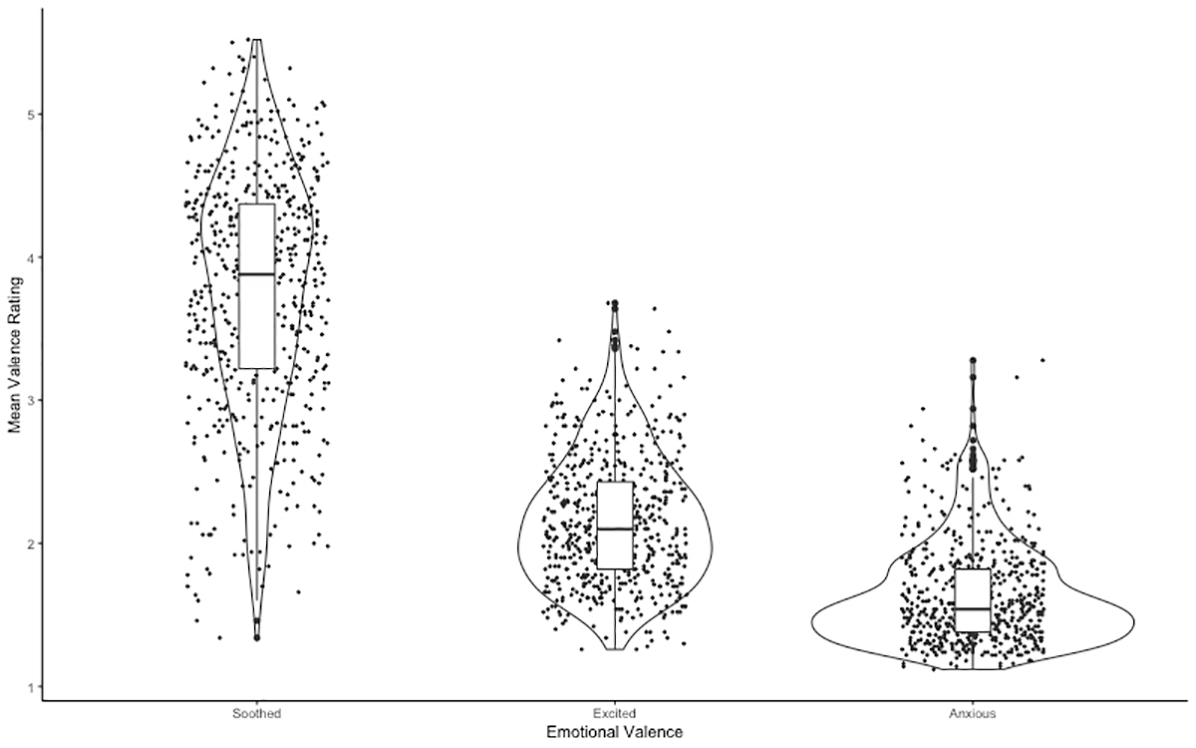
Box plot showing the median emotional valence (soothed, anxious, and excited) and inter quartile range from viewing the images. Error bars denote the upper and lower quartiles.

The results indicated that there was a significant main effect of emotional valence with a large effect size, *F*(1,81) = 510.134, *p* < 0.001, η ^2^ = .161. *Post hoc* comparisons indicated that ratings of soothed (M = 3.69, 95% CI: LL = 3.60, UL = 3.79) were significantly higher than ratings of anxious (M = 1.57, 95% CI: LL = 1.48, UL = 1.66, *p*_holm_ < 0.001) and ratings of excited (M = 2.09, 95% CI: LL = 2.00, UL = 2.18, *p*_holm_ < 0.001) in response to viewing the images. Additionally, ratings of anxious were significantly lower than ratings of excited ( *p*_holm_ < 0.001) from viewing the images ( Figure 1). As for set number effects, there was a significant emotional valence × set number interaction, *F*(1,81) = 3.767, *p* = 0.027, η ^2^ = 0.001. Therefore, three separate one-way ANOVAs were then performed to compare ratings of soothed, anxious, and excited between the 23 sets, with set number as the between-subject factor. Ratings of soothed was not found to be significantly different based on the set completed by participants, *F*(22,1129) = 1.05, *p* = 0.403, similarly for ratings of excited, *F*(22,1129) = 1.05, *p* = 0.395. However, there were significant differences in ratings of anxious depending on the set completed by participants with a small-medium effect size, *F*(22,1129) = 1.68, *p* = 0.025, η ^2^ = 0.032. This suggests that the set number completed by participants depending on their random allocation only affected the feelings of anxiety.

As hypothesised, the images were perceived to be significantly more soothing than anxious or exciting. To facilitate future research and clinical use of these images, we provided a ranking of all images from the most to the least soothing, based on their mean soothing rating. Table 2 depicts the descriptive data for the top 5 most soothing images ( Table 3; *Extended data* [ Project Soothe, 2023]) and the lowest 5 soothing images ( Table 4; *Extended data* [ Project Soothe, 2023]). A full spreadsheet with descriptive information for all images is included in the repository alongside the images (OSF, *Underlying data*).

**Table 2.** Mean, standard deviation, median, and interquartile range of emotional valence (soothed, anxious, and excited) from viewing the images (n = 50 per image). M: mean; SD: standard deviation; Mdn: median; IQR: interquartile range.

Image number	Image rank	Soothed				Anxious				Excited			
		*M*	*SD*	*Mdn*	*IQR*	*M*	*SD*	*Mdn*	*IQR*	*M*	*SD*	*Mdn*	*IQR*
569	1	5.52	1.47	6	2.75	1.34	0.80	1	0	2.42	1.59	2	2.75
472	2	5.5	1.49	6	2	1.34	0.82	1	0	2.9	1.90	3	3
419	3	5.4	1.64	6	2	1.24	0.77	1	0	2.28	1.88	1.5	2
410	3=	5.4	1.64	6	2	1.36	0.83	1	0	1.92	1.48	1	1.75
287	5	5.38	1.44	6	1	1.26	0.66	1	0	2.82	2.05	2	3
152	571	1.66	1.08	1	1	1.38	0.90	1	0	2.3	1.79	1	2
80	572	1.64	1.26	1	1	1.28	0.78	1	0	1.88	1.30	1	1
22	573	1.6	1.11	1	1	1.32	0.84	1	0	1.76	1.36	1	1
189	574	1.46	1.09	1	0	1.32	0.74	1	0	2.5	1.72	2	2
322	575	1.34	0.77	1	0	3.16	2.01	3	4	1.4	0.86	1	0

**Table 3.** Top 5 soothing images.

Rank	Image number	Image
1	569	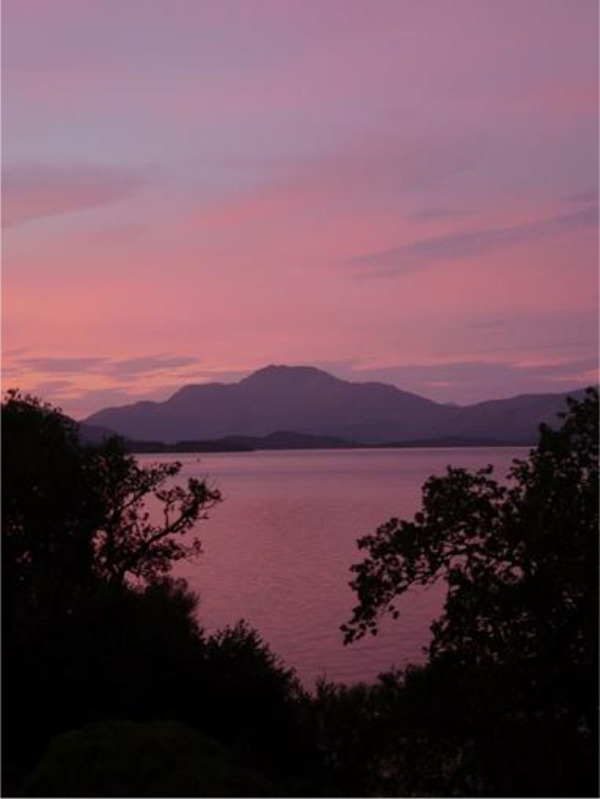
2	472	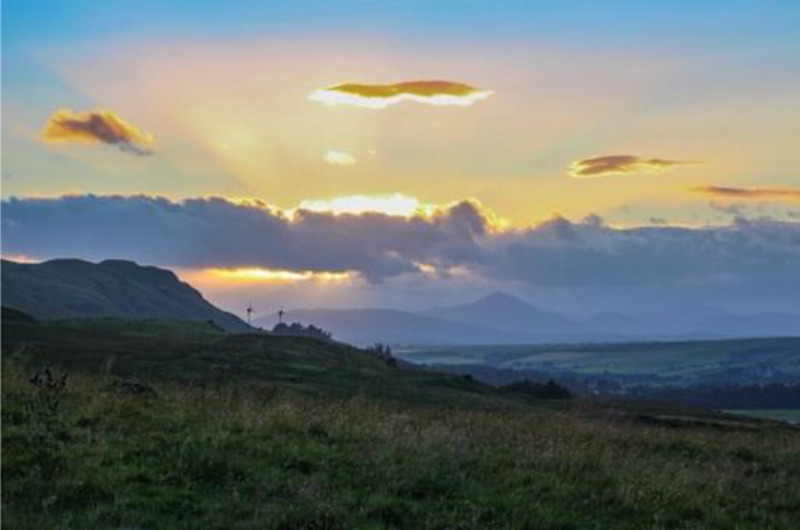
3	419	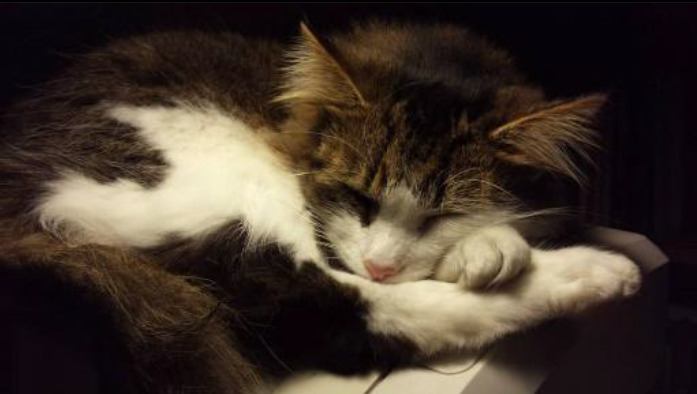
3=	410	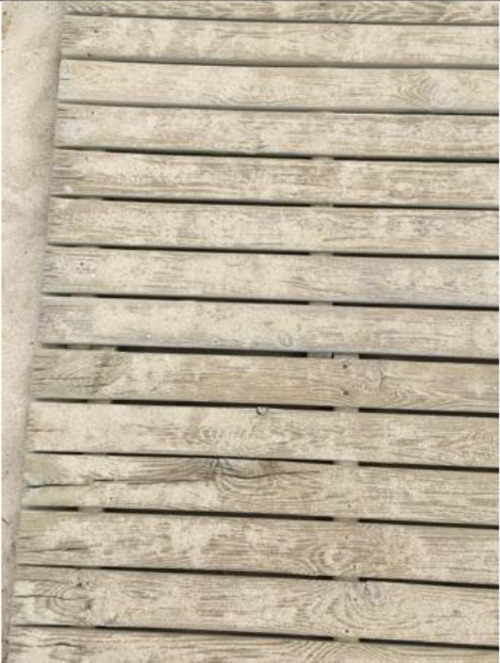
5	287	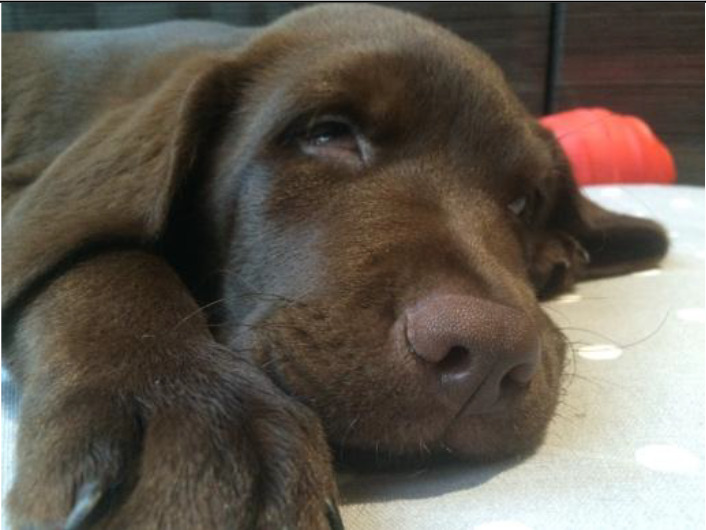

**Table 4.** Bottom 5 soothing images.

Rank	Image number	Image
571	152	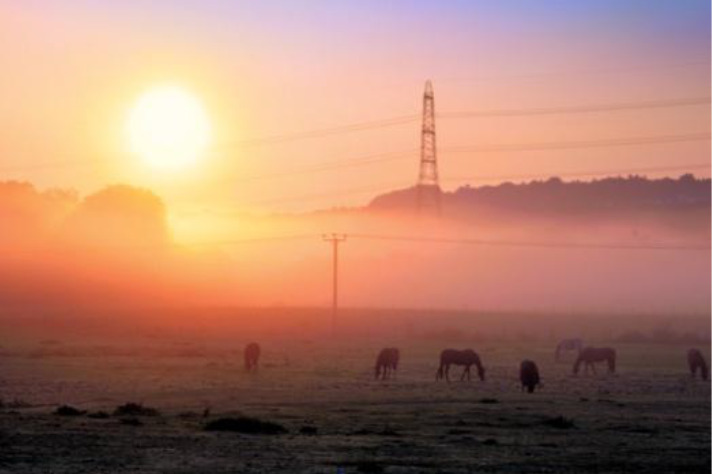
572	80	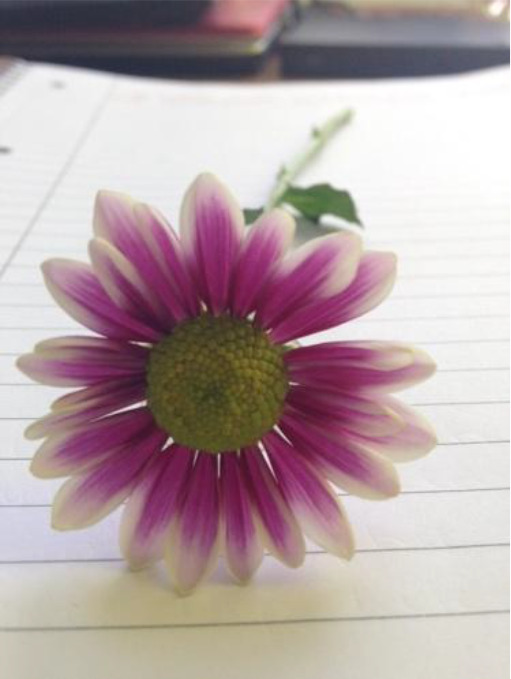
573	22	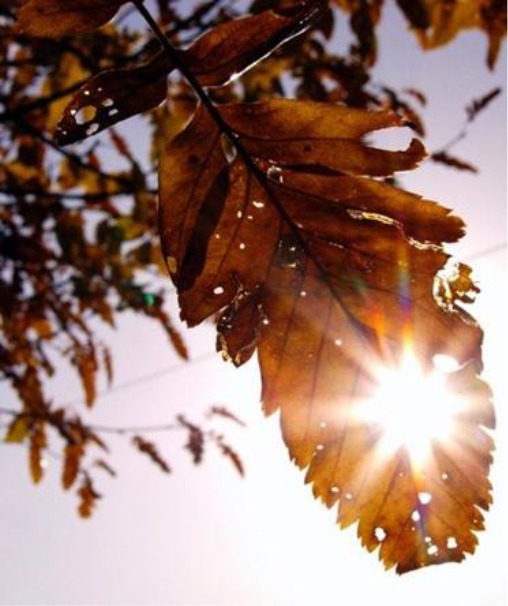
574	189	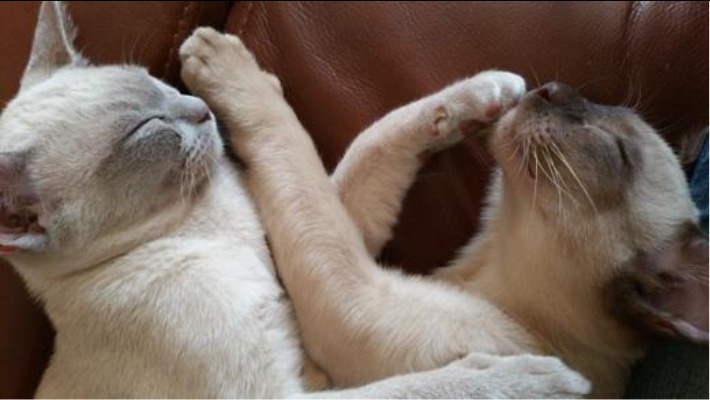
575	322	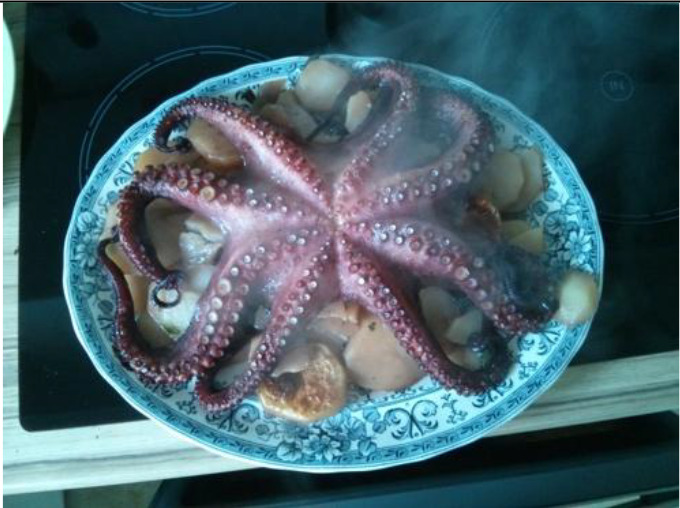

### Aim 2: Analyses to test if viewing the images induces positive mood changes

**Figure 2.**
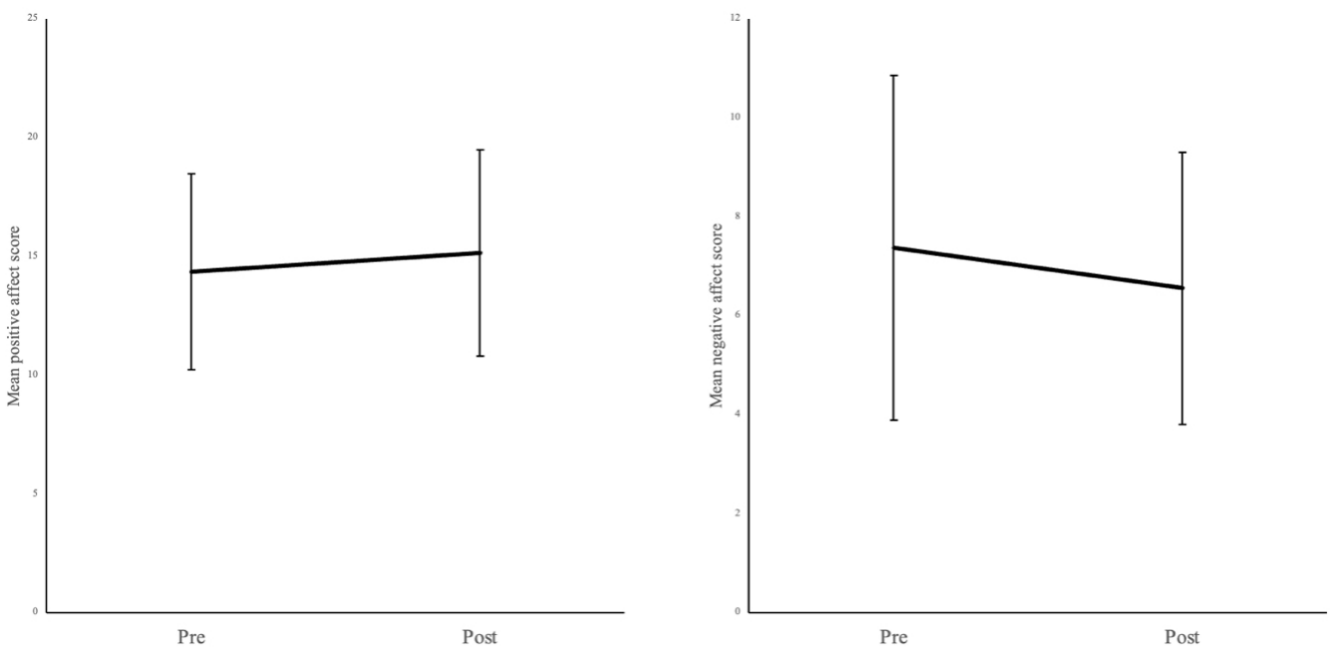
Mean positive and negative affect scores pre-viewing and post-viewing the soothing images when controlling for set number. Error bars denote standard deviation.

Based on the whole sample (N = 1152) and controlling for set number as a between-subject factor, results relating to positive affect indicated a significant main effect of time, with a very small effect size ( *F*(1,1129) = 80.93, *p* < 0.001, η ^2^ = 0.009), suggesting a significant increase in positive affect from pre-viewing ( *M* = 14.36, 95% CI: LL = 14.12, UL = 14.59) to post-viewing images ( *M* = 15.15, 95% CI: LL = 14.90, UL = 15.40, *p*_holm_ < 0.001) ( Figure 2). Additionally, there was no significant time × set number interaction ( *F*(22,1129) = 0.53, *p* = 0.963), indicating that the increase in positive affect was not influenced by set number.

Results relating to negative affect indicated a significant main effect of time with a small effect size ( *F*(1,1129) = 132.206, *p* < 0.001, η ^2^ = .016), suggesting a significant decrease in negative affect from pre-viewing ( *M* = 7.37, 95% CI: LL = 7.17, UL = 7.58) to post-viewing images ( *M* = 6.56, 95% CI: LL = 6.40, UL = 6.72) across the sample when controlling for set number ( *p*_holm_ < 0.001) ( Figure 2). Additionally, there was no significant interaction effect between time × set number ( *F*(22,1129) = 0.93, *p* = 0.553), indicating that the decrease in negative affect was not influenced by set number.

See Figure 2 for a graphical representation of the above main effects of time.

### Aim 3: Exploratory analyses to test if the findings varied with individual differences

Mean age of the participants was found to be significantly different between set number groups with a medium-large effect size ( *F*(22,1129) = 6.225, *p* < 0.001, η ^2^ = 0.108). There were no significant gender differences across set number groups (X ^2^(22, 1125) = 26.273, *p* = 0.240). As for depressive symptoms, mean PHQ-9 scores were found to be significantly different between set number groups with a small-medium effect size ( *F*(22,1129) = 1.674, *p* = 0.026, η ^2^ = 0.032) and the frequency of participants who were symptomatic/asymptomatic was also significantly different across set number groups (X ^2^(22, 1125) = 42.159, *p* = 0.006). Therefore, set number was controlled for in subsequent analyses relating to age and depressive symptoms.

***Age effects.*** In relation to emotional responses, we found a significant positive correlation between age and soothed ratings ( *r _s_* = 0.065, *p* = 0.026) and a significant negative correlation between age and anxious ratings ( *r _s_* = -0.222, *p* < 0.001), as well as age and excited ratings ( *r _s_* = -0.145, *p* < 0.001). These findings suggest that the older participants reported feeling more soothed and less anxious or excited from viewing the images than the younger participants.

As for mood changes across time, ANCOVAs with age added as a covariate and set number as a between-subject factor replicated previous analyses, finding a significant increase in positive affect ( *F*(1,1128) = 6.46, *p* = 0.011, η ^2^ = 0.000) and decrease in negative affect ( *F*(1,1128) = 51.00, *p* < 0.001, η ^2^ = 0.006) across time, but with very small effect sizes. Additionally, there was no significant time x age interaction ( *F*(1,1128) = 0.57, *p* = 0.450) for positive affect, indicating that the increase in positive affect showed no association with age. By contrast, there was a significant time x age interaction with a very small effect size ( *F*(1,1128) = 10.18, *p* = 0.001, η ^2^ = 0.001) for negative affect, indicating that the decrease in negative affect varied with age. There was no significant time × set number interaction for positive affect ( *p* > 0.05) or negative affect ( *p* > 0.05). *Post hoc* bivariate correlation analysis indicated a significant positive correlation between change in negative affect across time and age ( *r _s_* = 0.100, *p* < 0.001), driven by older individuals reporting greater reduction in negative affect from viewing the images than younger individuals.

***Gender effects.*** In relation to emotional responses, we found no significant gender differences for ratings of soothed ( *Z*(1152) = 108786.5, *p* = 0.270), anxious ( *Z*(1152) = 100078.5, *p* = 0.384), or excited ( *Z*(1152) = 97161.0, *p* = 0.126), suggesting that emotional responses were not associated with gender.

Regarding mood changes across times, repeated-measure ANOVAs with gender entered as a between-subject factor replicated earlier analyses, suggesting a significant increase in positive affect with a very small effect size ( *F*(1,1123) = 49.13, *p* < 0.001, η ^2^ = 0.005) and decrease in negative affect ( *F*(1,1123) = 86.86, *p* < 0.001, η ^2^ = 0.011) across time. Additionally, there was no significant time × gender interaction for positive affect ( *F*(1,1123) = 0.16, *p* = 0.694) or negative affect ( *F*(1,1123) = 0.00, *p* = 0.979), suggesting that the current findings are independent of the effects of gender.

***Depression symptom effects.*** In relation to emotional responses, we found a significant negative correlation between PHQ-9 score and soothed ratings with a small-medium effect size ( *r _s_* = -0.069, *p* = 0.019) and a significant positive correlation between PHQ-9 score and anxious ratings with a large effect size ( *r _s_* = 0.292, *p* < 0.001), and excited ratings with a small-medium effect size ( *r _s_* = 0.062, *p* = 0.036). These findings suggest that individuals with higher levels of depressive symptoms perceived the images as less soothed and more anxious and excited than individuals with lower levels of depressive symptoms.

As for mood changes across time, repeated measures ANOVAs with depression symptom level and set number entered as a between-subject factors replicated earlier analyses suggesting a significant increase in positive affect with a very small effect size ( *F*(1,1106) = 78.29, *p* < 0.001, η ^2^ = 0.008) and decrease in negative affect with a small effect size ( *F*(1,1106) = 111.75, *p* < 0.001, η ^2^ = 0.014) across time. There was no significant interaction involving set number for positive affect ( *F*(22,1106) = 0.78, *p* = 0.758) or negative affect ( *F*(22,1106) = 0.89, *p* = 0.602). Additionally, there was no significant interaction effect between time × depression level ( *F*(1,1106) = 0.77, *p* = 0.381) for positive affect. However, there was a significant interaction effect between time × depression level with a very small effect size ( *F*(1,1106) = 35.24, *p* < 0.001, η ^2^ = 0.004) for negative affect, indicating that the decrease in negative affect was greater for symptomatic participants than asymptomatic participants ( *p*_holm_ < 0.001).

Repeated measures ANCOVAs indicated a significant increase in positive affect with a very small effect size ( *F*(1,1128) = 21.64, *p* < 0.001, η ^2^ = 0.002) and a decrease in negative affect with a very small effect size ( *F*(1,1128) = 7.03, *p* = 0.008, η ^2^ = 0.000) across time. There were no significant interactions involving set number for positive affect ( *p* > 0.05) or negative affect ( *p* > 0.05). Additionally, there was no significant time × PHQ-9 interaction ( *F*(1,1128) = 2.34, *p* = 0.126) in relation to positive affect. However, there was a significant time × PHQ-9 interaction with a very small effect size ( *F*(1,1128) = 41.49, *p* < 0.001, η ^2^ = 0.005), indicating that decreases in negative affect across time was associated with PHQ-9 score.

## Discussion

Project Soothe was designed to collect images that can be used in research and therapies; in particular, it focused on soothing images in line with Gilbert’s (2009) model of emotion. The current study was conducted to evaluate if the images collected from members of the public generate soothing effects and positive mood changes. Our key findings provide preliminary evidence that the collected images, gathered using an innovative Citizen Science method, were indeed perceived to be more soothing as intended than the contrasting emotional responses (namely anxious and excited) proposed in the theoretical model. Although we observed set number to be associated with anxious ratings, suggesting differences depending on the set of images participants were allocated to view, this is likely to have been driven by the fact that relatively few participants experiencing anxiety in response to images, and only specific images in specific sets inducing these feelings. In line with Open Science framework and to facilitate future use of images by researchers and practitioners, based on findings of this study, we have ranked the images from the most soothing to the least soothing. The images alongside the descriptive statistics and ranking of these images can now be found in the data repository ( *OSF, Underlying data*) as a freely available and accessible resource.

Although mood improvements from viewing the images was consistent throughout our analyses, our findings indicated that the strength of effects may vary with individual differences. Our findings suggest that effects were consistent across male and female genders, although we were not able to examine effects outside the gender binary due to small sample sizes. However, there were some differences attributed to age and depressive symptoms. Firstly, our findings indicate that age is positively associated with the perception of the images to be more soothing and a greater reduction in negative affect after viewing 25 images. This may be due to a positivity effect across development, in which older adults have been observed to be more likely to remember and attend to positive stimuli, compared to younger ages who appeared to be more biased towards negative stimuli ( Carstensen & DeLiema, 2018). However, as age in our study was skewed towards younger adults, future work should seek to understand if effects differ across different age ranges. Secondly, our results preliminarily suggest that individuals with and without depressive symptoms can benefit from viewing soothing images, although the effects seem to be stronger in those with lower or no symptoms. This is in line with previous research that suggests depression is associated with reduced responsiveness to positive stimuli ( Dunn *et al.*, 2004; Wilson *et al.*, 2018). It may also suggest that individuals with higher level of depressive symptoms would require a higher ‘dose’ ( *e.g.*, more images or longer duration of exposure) to experience the same level of mood effects. Taken together, viewing Project Soothe images appears to have an overall effect for inducing soothing feelings and positively impacting mood. It should however be noted that these individual differences, though statistically significant, were only of small effect sizes and hence the observations here are only tentative. This may be due to the fact that as this study aimed to evaluate the image bank, participants would have been exposed to a mixture of images with differing soothing effects. Additionally, scores on the PANAS-SF indicate that the participants were generally in a positive mood state pre-viewing the images, scoring on average 14.36 for positive affect and 7.37 for negative affect (possible range for each subscale: 5 – 25). Future work can now utilise the newly established most and least soothing images, and examine in clinical populations, to test if results are replicated with greater effect sizes.

Furthermore, although viewing Project Soothe images was found to have a positive influence on mood consistently in our results, mean ratings of soothed in response to the individual images were lower and had more variability between individuals than anticipated. This may suggest that the images may not be inducing positive mood changes through stimulating the soothing system as theoretically predicted; instead, the mood changes may perhaps be associated with other underlying mechanisms and corresponding positive emotions. For instance, as pre- and post-affect was measured using the I-PANAS-SF ( Karim *et al.*, 2011), this could suggest that the increases in positive affect from viewing the images are due to other emotional mechanisms as associated with ‘active’, ‘determined’, ‘attentive’, ‘inspired’, and ‘alert’ (which were the items of the scale). Alternatively, viewing the images may be subject to a ‘dosing effect’ whereby soothing effects resulting from the images is cumulative, thus images may not be perceived as soothing in isolation, but can stimulate the soothing system when viewed collectively. Due to these questions raised, we have ranked the images and reported the 25 ^th^ percentile in the full spreadsheet (available via OSF) to help clinicians and researchers identify the images that are more commonly perceived as soothing. This may be particularly helpful for clinicians at the start of intervention before individual preferences could be explored. It would also be beneficial for future research to understand more about the mechanisms of change that underlie the positive effects of the Project Soothe images observed here. Of note, since the current study, we have conducted further studies to identify how the effects of Project Soothe images could be boosted and our preliminary results suggest that combining them with mindful breathing exercises could offer additional mood benefits (Grace and Chan, under review).

Encouragingly, our findings suggest that viewing 25 images randomly selected from the collection led to a significant increase in positive affect and decrease in negative affect, which has various implications. Mentally-generated soothing images have been routinely used in CFT ( Gilbert, 2009) and our findings extended the literature by suggesting that externally presented soothing images can also elicit positive mood states. As a pilot study, we evaluated the effects using a simple pre- and post-design, thus limiting our ability to rule out the possibility that the positive outcomes were due to factors unrelated to viewing the soothing images. Nevertheless, it is encouraging to note that our findings were consistent with our recent study that included a comparison group and extended outcome measures ( Witten *et al.*, 2023) and, together, suggest that upon further research Project Soothe images have potential to be developed as a self-help tool or be integrated into relevant existing therapeutic approaches. The fact that the images were submitted by members of the public using a Citizen Science methodology further suggests the potential of encouraging individuals and communities to share soothing images for wellbeing purposes. As the beneficial effect was found to be greater in those who do not need a clinical intervention (lower depressive symptoms), these images could be used as a beneficial preventive strategy. Indeed, it is encouraging to note that our research group has recently started collaborating with a local council in exploring how Project Soothe could be used in the context of social prescribing and as a preventative strategy in supporting mental wellbeing in primary and secondary schools. In these new initiatives, the images could be tested further in non-clinical groups and with children and young people, to examine their utility for this purpose.

### Limitations and future directions

In this study, as acknowledged above, we had only compared mood states from before to after viewing the images using brief mood measures; hence we are unable to rule out other factors (such as distraction) that might have contributed to the changes observed. However, we have now conducted further studies with comparison groups and extended outcome measures. Furthermore, we positioned the depression scale towards the end of the study to reduce the possibility that filling in a mood questionnaire may create a temporary mood induction effect on participants. However, it is also possible that participants’ improved mood states may have influenced their self-reported depression scores. Although the PHQ-9 asks about experiences over the past two weeks, and hence should reflect participants’ current level of depressive symptoms without being influenced by the order of administration within a single sitting, our findings regarding the role of depressive symptoms should nonetheless be interpreted with caution. Furthermore, future research should aim to examine the effect of these images in a clinical population to further inform the feasibility and potential of applying these images therapeutically. Additionally, as this study was our first attempt in evaluating the effects of the bank of images, we did not have the relevant data (e.g. effect sizes) to perform an a priori sample size calculation, but the reported effect sizes can be used in future research. Finally, the magnitude of the mean changes in positive and negative affect are relatively small and as we have only so far examined state affect, it is not yet known whether the images can be used to induce longer-term beneficial effects beyond temporary changes in mood states. As such, to examine this, future work should seek to employ longitudinal designs, that investigate effects from repeated image exposure as well as follow-up measures of mood. Further, future work should also seek to examine effects on other mental health symptoms, such as anxiety. To facilitate further research development, we have created a ranked list that highlights the most soothing images and made this data and the images freely available in an open access platform.

## Conclusions

The results of this study and database of soothing images open interesting future directions for both experimental and clinical research. The current evaluation of the Project Soothe images suggests that they are viable external stimuli to bring about soothing feelings, increase positive affect and reduce negative affect. These effects appear to be stronger in older people and those with lower depressive symptoms. These preliminary findings provide proof-of-concept evidence that, upon further research and evaluation, Project Soothe images have the potential to be developed as therapeutic resources. To facilitate further research and intervention development, the images are now freely available in the data repository (OSF) that can be used by researchers and practitioners.

## Data Availability

Open Science Framework: Project Soothe: A pilot study evaluating the mood effects of soothing images collected using a citizen science approach, https://doi.org/10.17605/OSF.IO/2EGZT ( Project Soothe, 2023) This project contains the following underlying data: ProjectSootheImageRanking.xls This project contains the following extended data: Extended data Data are available under the terms of the Creative Commons Attribution 4.0 International license (CC-BY 4.0).
