# Supplementary material for: Project Soothe: A pilot study evaluating the mood effects of soothing images collected using a citizen science approach
Source: Wellcome Open Res. 2024 Mar 1;8:218. Originally published 2023 May 12. [Version 2] doi: 10.12688/wellcomeopenres.18950.2 (PMC11474226; doi:10.12688/wellcomeopenres.18950.2)
Supplement: Peer review history

# Reviewer report: Reviewer response for version 2

**DOI:** 10.21956/wellcomeopenres.23317.r104080

**Published:** 2024-10-14

**Authors:** Stephanie Burnett Heyes

**Affiliations:** 1 University of Birmingham,, Birmingham, UK

- This study was conducted using a citizen science approach to gather and rate the affective impact of images. The sample is large and well characterised. The aims/hypotheses are clear and mostly well grounded theoretically (cf. compassion focused therapy, mental imagery). The data collection and analysis methods are described well and the results are interpreted appropriately. Data are available online (i.e. composite scores for each image across participants). The findings are novel and interesting, e.g. differential impact on affect based on PHQ depression symptom scale, and selective impact on feelings of soothe vs. anxiety/excitement, consistent with study aims. Findings are interpreted appropriately in the main, including with suggestions for developing the evidence base around mechanisms, and for how to use the images. 
- I would like to see a little more information about the predicted effects of age and the analysis. Is there reason to expect age effects, and should these be linear? 
- I object to the statement based on reduced effect in high PHQ scorers that the images "could be used as a beneficial preventive strategy". I would say more evidence is needed to make this claim. 

Is the work clearly and accurately presented and does it cite the current literature?

Yes

If applicable, is the statistical analysis and its interpretation appropriate?

Yes

Are all the source data underlying the results available to ensure full reproducibility?

Partly

Is the study design appropriate and is the work technically sound?

Yes

Are the conclusions drawn adequately supported by the results?

Yes

Are sufficient details of methods and analysis provided to allow replication by others?

Yes

Reviewer Expertise:

developmental psychology, experimental psychopathology

I confirm that I have read this submission and believe that I have an appropriate level of expertise to confirm that it is of an acceptable scientific standard, however I have significant reservations, as outlined above.

# Reviewer report: Reviewer response for version 2

**DOI:** 10.21956/wellcomeopenres.23317.r75851

**Published:** 2024-08-26

**Authors:** Julie Lin Ji

**Affiliations:** 1 School of Psychology, University of Plymouth, Plymouth, England, UK

The authors have addressed my comments and the manuscript is now more coherent and clear. No further comments.

Is the work clearly and accurately presented and does it cite the current literature?

Yes

If applicable, is the statistical analysis and its interpretation appropriate?

Partly

Are all the source data underlying the results available to ensure full reproducibility?

Yes

Is the study design appropriate and is the work technically sound?

Yes

Are the conclusions drawn adequately supported by the results?

Partly

Are sufficient details of methods and analysis provided to allow replication by others?

Partly

Reviewer Expertise:

Cognition and emotion; information-processing biases; mood and anxiety; mental imagery

I confirm that I have read this submission and believe that I have an appropriate level of expertise to confirm that it is of an acceptable scientific standard.

# Reviewer report: Reviewer response for version 1

**DOI:** 10.21956/wellcomeopenres.21010.r59018

**Published:** 2023-07-17

**Authors:** Julie Lin Ji

**Affiliations:** 1 School of Psychology, University of Plymouth, Plymouth, England, UK

This paper reports on emotion ratings and mood impacts of the Project Soothe image dataset. This study is important in that it aims to evaluate whether a) the Project Soothe images are soothing; and b) whether viewing such images have beneficial mood impacts. The manuscript is clearly written and the study is straight forward and well conducted. I have the following queries that I consider important to address in order to help readers understand the study and interpret the implications of its findings. 

**Key query:**

 My main query concerns the finding that viewing 25 Project Soothe images led to significant increases in positive affect and significant decreases in negative affect. Although the finding is statistically significant, this should not be interpreted out of context, as the sample size was large (N = 1152), and the magnitude of score change was miniscule. I.e. mean scores in positive affect increased from 14.36 to 15.15, and the negative affect scores decreased from 7.37 to 6.56, as measured by the PANAS-SF and depicted in Figure 2.

- It would seem important not to only focus on the statistical significance of this effect, but rather to a) report and focus interpretation on effect size; b) to contextualise the rating scores in relation to the PANAS-SF rating scale, i.e. what does a score of 15.15 mean? ; c) discuss the size of the emotion change and how it relates to the ultimate purpose of Project Soothe, i.e. as a clinical tool to increase positive affect and reduce negative affect.

**Other queries:**

 Introduction: The introduction is brief and succinct, which is appropriate given the focus of the study was to evaluate the image dataset.

- I was slightly surprised to see the first half of the introduction being devoted to mental imagery, as the study itself does not test any hypotheses concerning mental imagery. Please consider moving this content to later parts of the introduction, as a secondary focus.
- Relatedly, although the purpose of the Project Soothe project has been described in previous papers, it is worth considering opening the introduction with this information, most of which are contained in the final paragraph.

Method:

- Sample size – can the authors please explain how the total sample size was arrived at, and how the set sample size of N = 50 was arrived at.
- Image ratings task – it would be good to see information on a) exact instructions given to participants; b) how long participants were given to rate each image, and whether viewing time was standardised/constrained or not.
- PHQ depression scale – please can the authors clarify a) why the PHQ scores were categorised into binary bins (Symptomatic vs. Asymptomatic), as it is also analysed as a continuous score in the correlational analyses with emotion ratings; and b) how this categorisation was done and the justification for this.

Is the work clearly and accurately presented and does it cite the current literature?

Yes

If applicable, is the statistical analysis and its interpretation appropriate?

Partly

Are all the source data underlying the results available to ensure full reproducibility?

Yes

Is the study design appropriate and is the work technically sound?

Yes

Are the conclusions drawn adequately supported by the results?

Partly

Are sufficient details of methods and analysis provided to allow replication by others?

Partly

Reviewer Expertise:

Cognition and emotion; information-processing biases; mood and anxiety; mental imagery

I confirm that I have read this submission and believe that I have an appropriate level of expertise to confirm that it is of an acceptable scientific standard, however I have significant reservations, as outlined above.

# Response

**Published:** 2024-02-12

**Authors:** Keren MacLennan

**Affiliations:** Durham University, UK

*Thank you for taking the time to review our paper and for your very helpful comments. We have submitted a revised manuscript and have addressed your comments point by point below.* Key query: My main query concerns the finding that viewing 25 Project Soothe images led to significant increases in positive affect and significant decreases in negative affect. Although the finding is statistically significant, this should not be interpreted out of context, as the sample size was large (N = 1152), and the magnitude of score change was miniscule. I.e. mean scores in positive affect increased from 14.36 to 15.15, and the negative affect scores decreased from 7.37 to 6.56, as measured by the PANAS-SF and depicted in Figure 2. It would seem important not to only focus on the statistical significance of this effect, but rather to a) report and focus interpretation on effect size; b) to contextualise the rating scores in relation to the PANAS-SF rating scale, i.e. what does a score of 15.15 mean? ; c) discuss the size of the emotion change and how it relates to the ultimate purpose of Project Soothe, i.e. as a clinical tool to increase positive affect and reduce negative affect.

- *We have more strongly reported the effect sizes in the results section and have added further discussion around the interpretation of the effect sizes. As this was an initial evaluation of the entire image bank, it is understandable that effect sizes may not be large. Especially as participants would have been exposed to images with varying soothing effects. The images rated as being most soothing can now be tested further in future work, perhaps in comparison to least soothing, to see if these effects are replicated with larger effect sizes. We have also contextualised the PANAS-SF scores to highlight that we further need to understand effects in clinical populations. We have added additional discussion of this to the paper: “Taken together, viewing Project Soothe images appears to have an overall effect for inducing soothing feelings and positively impacting mood. It should however be noted that these individual differences, though statistically significant, were only of small effect sizes and hence the observations here are only tentative. This may be due to the fact that as this study aimed to evaluate the image bank, participants would have been exposed to a mixture of images with differing soothing effects. Additionally, scores on the PANAS-SF indicate that the participants were generally in a positive mood state pre-viewing the images, scoring on average 14.36 for positive affect and 7.37 for negative affect (possible range for each subscale: 5 – 25). Future work can now utilise the newly established most and least soothing images, and examine in clinical populations, to test if results are replicated with greater effect sizes.”*

Other queries: Introduction: The introduction is brief and succinct, which is appropriate given the focus of the study was to evaluate the image dataset. I was slightly surprised to see the first half of the introduction being devoted to mental imagery, as the study itself does not test any hypotheses concerning mental imagery. Please consider moving this content to later parts of the introduction, as a secondary focus. Relatedly, although the purpose of the Project Soothe project has been described in previous papers, it is worth considering opening the introduction with this information, most of which are contained in the final paragraph.

- *To address these points, we have reordered the focus of the introduction so that we start by describing Project Soothe and theory underpinning this, and then we describe the links to mental imagery approaches used therapeutically.*

Method: Sample size – can the authors please explain how the total sample size was arrived at, and how the set sample size of N = 50 was arrived at.

- *We have included this in the limitations as it was an initial validation study and we did not have information related to relevant effect sizes: “Additionally, as this study was our first attempt in evaluating the effects of the bank of images, we did not have the relevant data (e.g. effect sizes) to perform an a priori sample size calculation, but the reported effect sizes can be used in future research.”*

Image ratings task – it would be good to see information on a) exact instructions given to participants; b) how long participants were given to rate each image, and whether viewing time was standardised/constrained or not.

- *We have now included this information: “The instructions as the commencement of this task were: “You will now be shown a collection of images for you to evaluate. For each image, please take a moment to consider how soothed, anxious and excited the image makes you feel. Then, use the scales under each image to rate your feelings.” Participants were able to view the images an unconstrained amount of time before providing their ratings.”*

PHQ depression scale – please can the authors clarify a) why the PHQ scores were categorised into binary bins (Symptomatic vs. Asymptomatic), as it is also analysed as a continuous score in the correlational analyses with emotion ratings; and b) how this categorisation was done and the justification for this.

- *We have added further information in the Analysis section to clarify this: We collapsed the categories this way due to the data being from a non-clinical sample and therefore higher levels of symptom severity did not contain sufficient cases to be statistically powered. To further examine the potential relationship with level of depressive symptoms, we supplemented the above by conducting two separate repeated measures ANCOVAs to test if changes in positive and negative affect across time (two levels: pre-viewing images and post-viewing images) were replicated when set number was entered as a between-subject factor and the PHQ-9 scores were entered as a covariate. The utility of examining depressive symptoms from a categorical and continuous perspective is that the PHQ-9 is a measure often used in clinical practice (e.g. IAPT service), where categories are used. Therefore, conducting analyses using categories of symptomatic vs asymptomatic would provide practitioners information that can be more easily interpreted in clinical contexts. At the same time, by categorising scores and collapsing groups like this, we would not be able to provide more precise findings regarding the association with the exact level of depressive symptoms. Therefore, we also performed analyses where PHQ scores were used as a continuous variable.*

# Reviewer report: Reviewer response for version 1

**DOI:** 10.21956/wellcomeopenres.21010.r57346

**Published:** 2023-06-01

**Authors:** Jerica Radez

**Affiliations:** 1 Oxford Institute of Clinical Psychology Training and Research, Medical Sciences Division, University of Oxford, Oxford, England, UK

This study is very interesting and novel, and I think that it will appeal to a wide range of audiences. The study was conducted using rigorous methodological approaches.

 I have only a few minor comments/suggestions.

 Introduction: Good overview of the literature and well-written.

- I am finding the last paragraph a bit difficult to read. It starts with the present, and then describes your previous work that informed the current study. I wonder if it would read easier if you would re-structure it (i.e., starting with your groundwork that informed your current study)? I would also suggest splitting the last paragraph in two paragraphs – first one describing your previous work/why the need for your current study and the second paragraph specify this study’s aims.

Methods: In general, clear and well-written. It’s also great to see the project published on the OSF.

- I would like to see a bit more info on how participants were approached (e.g., which social media; what was a recruitment window).
- Was the sample size pre-determined (a priori). If so, can you please add the details.
- Also, I would find it helpful to know more about the age distribution (e.g., % of people in different age groups or perhaps use Median instead of Mean) – especially given that the age was an important covariate and that the study was conducted via social media/websites, which generally appeal more to younger generations.
- Analyses are very detailed, which is good. However, I have a few questions/comments to make this section a bit clearer – is there any reason that the whole sample was included for aim 2 and subsets of 50 for aim 1? Also, for aim 3, please specify which variables were not normally distributed (especially as you then continue with using ANOVAs, which are parametric tests).

Results: Results are clear and well-structured.

 Discussion: The narrative in discussion follows nicely from previous sections. I have a couple of comments and suggestions to make this section a bit ‘stronger’:

- Third paragraph – you talk about ‘older adults’ – see my comment above – it’s important to specify what do you mean by this (i.e., age range or people over certain age).
- I would like to see a paragraph about implications – i.e., beyond clinical work. I think that there is a line of argument around thinking about using compassionate images as a preventive strategy (perhaps in younger people/schools), given that the effects seem to be more pronounced for those who do not need a clinical intervention (i.e., those with low scores on PHQ-9). Also, it would be good to expand the paragraph on future research – e.g., repeated exposure to images and follow-up measures of mood; measuring different symptoms (not just depressive symptoms) etc.

Is the work clearly and accurately presented and does it cite the current literature?

Yes

If applicable, is the statistical analysis and its interpretation appropriate?

Yes

Are all the source data underlying the results available to ensure full reproducibility?

Yes

Is the study design appropriate and is the work technically sound?

Yes

Are the conclusions drawn adequately supported by the results?

Yes

Are sufficient details of methods and analysis provided to allow replication by others?

Yes

Reviewer Expertise:

Child and adolescent mental health; clinical psychology; early intervention in psychosis/ARMS

I confirm that I have read this submission and believe that I have an appropriate level of expertise to confirm that it is of an acceptable scientific standard, however I have significant reservations, as outlined above.

# Response

**Published:** 2024-02-12

**Authors:** Keren MacLennan

**Affiliations:** Durham University, UK

Authors comments are in red. Thank you for taking the time to review our manuscript and for your helpful comments. We have re-submitted a revised manuscript and addressed your comments point by point below. Introduction: Good overview of the literature and well-written. I am finding the last paragraph a bit difficult to read. It starts with the present, and then describes your previous work that informed the current study. I wonder if it would read easier if you would re-structure it (i.e., starting with your groundwork that informed your current study)? I would also suggest splitting the last paragraph in two paragraphs – first one describing your previous work/why the need for your current study and the second paragraph specify this study’s aims.

- We have now taken your feedback onboard. Specifically, we have reordered the focus of the introduction in alignment with Reviewer 2 (JLJ) so that we start by describing Project Soothe and theory underpinning this, and then we describe the links to mental imagery approaches used therapeutically. The final paragraph is now only describing this study’s aims. We hope the re-structuring of the introduction has improved clarity in line with both reviewers’ comments.

Methods: In general, clear and well-written. It’s also great to see the project published on the OSF. I would like to see a bit more info on how participants were approached (e.g., which social media; what was a recruitment window).

- We have included information of the social media sites that were used for recruitment (i.e. Twitter, Facebook). We have also added details about the recruitment window for this study: “Participants were recruited via social media (Twitter, Facebook) and listed on websites for Citizen Science projects. Recruitment and data collection for 12 months, between May 2017 and May 2018.”

Was the sample size pre-determined (a priori). If so, can you please add the details.

- This study was our first attempt in evaluating the effects of the bank of images we collected. As such, we did not have the relevant data (e.g. effect sizes) to perform a priori sample size calculation. We have now acknowledged this as a limitation of the study in Discussion.

Also, I would find it helpful to know more about the age distribution (e.g., % of people in different age groups or perhaps use Median instead of Mean) – especially given that the age was an important covariate and that the study was conducted via social media/websites, which generally appeal more to younger generations.

- Median age distribution has now been included alongside the mean.

Analyses are very detailed, which is good. However, I have a few questions/comments to make this section a bit clearer – is there any reason that the whole sample was included for aim 2 and subsets of 50 for aim 1? Also, for aim 3, please specify which variables were not normally distributed (especially as you then continue with using ANOVAs, which are parametric tests).

- As outlined in the materials section, each image was viewed by 50 participants as they were randomly allocated to an image set. Therefore, aim 1 has an n = 50 for each image. Whereas aim 2, which is non-image specific, included the full sample. We have now elaborated this further to clarify this in the analysis section: “The above analysis was based on a sample size of 50 as it relates to the data available for each image ( i.e., the total number of participants assigned to each image set).”
- We have also now specified the variables that were not normally distributed, namely age and depressive symptoms. It was deemed appropriate to use parametric tests as ANOVA is robust to non-normally distributed data and the sample size was sufficiently large.

Discussion: The narrative in discussion follows nicely from previous sections. I have a couple of comments and suggestions to make this section a bit ‘stronger’: Third paragraph – you talk about ‘older adults’ – see my comment above – it’s important to specify what do you mean by this (i.e., age range or people over certain age).

- We have reworded this to more accurately  reflect the findings of the study. Age was simply a covariate in this study and future work should explore the effect of age further. “Firstly, our findings indicate that age is positively associated with the perception of the images to be more soothing and a greater reduction in negative affect after viewing 25 images. This may be due to a positivity effect across development, in which older adults have been observed to be more likely to remember and attend to positive stimuli, compared to younger ages who appeared to be more biased towards negative stimuli ( Carstensen & DeLiema, 2018). However, as age in our study was skewed towards younger adults, future work should seek to understand if effects differ across different age ranges.”

I would like to see a paragraph about implications – i.e., beyond clinical work. I think that there is a line of argument around thinking about using compassionate images as a preventive strategy (perhaps in younger people/schools), given that the effects seem to be more pronounced for those who do not need a clinical intervention (i.e., those with low scores on PHQ-9). Also, it would be good to expand the paragraph on future research – e.g., repeated exposure to images and follow-up measures of mood; measuring different symptoms (not just depressive symptoms) etc.

- We have moved down the second paragraph that discusses implications and have expanded to include reflections beyond clinical work: “Encouragingly, our findings suggest that viewing 25 images randomly selected from the collection led to a significant increase in positive affect and decrease in negative affect, which has various implications. Mentally-generated soothing images have been routinely used in CFT ( Gilbert, 2009) and our findings extended the literature by suggesting that externally presented soothing images can also elicit positive mood states. As a pilot study, we evaluated the effects using a simple pre- and post-design, thus limiting our ability to rule out the possibility that the positive outcomes were due to factors unrelated to viewing the soothing images. Nevertheless, it is encouraging to note that our findings were consistent with our recent study that included a comparison group and extended outcome measures ( Witten et al., 2023) and, together, suggest that upon further research Project Soothe images have potential to be developed as a self-help tool or be integrated into relevant existing therapeutic approaches. The fact that the images were submitted by members of the public using a Citizen Science methodology further suggests the potential of encouraging individuals and communities to share soothing images for wellbeing purposes. As the beneficial effect was found to be greater in those who do not need a clinical intervention (lower depressive symptoms), these images could be used as a beneficial preventive strategy. Indeed it is encouraging to note that our research group has recently started collaborating with a local council in exploring how Project Soothe could be used in the context of social prescribing and as a preventative strategy in supporting mental wellbeing in primary and secondary schools. In these new initiatives, the images would be tested further in non-clinical groups and with children and young people, to examine their utility for this purpose.”
- We have also expanded on the future directions to emphasise the type of future work which is needed: “To examine this, future work should seek to employ longitudinal designs, that investigate effects from repeated image exposure as well as follow-up measures of mood. Further, future work should also seek to examine effects on other mental health symptoms, such as anxiety.”
